# Research networking and the role of the medical librarian

**DOI:** 10.5195/jmla.2024.1887

**Published:** 2024-04-01

**Authors:** Robyn Reed, Matthew J. Eyer, Megan M. Young, Sarah K. Bronson

**Affiliations:** 1 rbr11@psu.edu, Harrell Health Sciences Library, Penn State College of Medicine, Hershey, PA; 2 mje151@psu.edu, Multimedia Specialist (Web Content, Design and Development), Office of Strategic Marketing and Communications, Penn State College of Medicine, Hershey, PA; 3 mmy10@psu.edu, Assistant Director of Research Development and Assistant Professor of Pharmacology, Penn State College of Medicine, Hershey, PA; 4 mmy10@psu.edu, (former) Associate Dean of Interdisciplinary Research, Director of Research Development and Associate Professor of Cellular and Molecular Physiology, (current) Associate Professor Emeritus, Penn State College of Medicine, Hershey, PA

**Keywords:** Research networking, collaboration, biomedical research

## Abstract

Medical librarians work collaboratively across all units and missions of academic medical centers. One area where librarians can provide key expertise is in the building and maintenance of Research Information Management Systems (RIMS). At Penn State, the RIMS implementation team has included a medical librarian, research administrators and marketing staff from the College of Medicine (CoM) since its inception in 2016. As our peer institutions implemented or expanded their own RIMS systems, the CoM team has responded to their questions regarding details about the Penn State RIMS instance. The goal of this commentary is to describe how the CoM team has worked collaboratively within Penn State to address questions related to research output, with special emphasis on details pertaining to questions from other institutions.

## BACKGROUND

Research networking systems in academic institutions may be called Current Research Information Systems (CRIS), Research Information Management Systems (RIMS), Research Networking Systems (RNS) or other similar terms [1]. The motivations for building a research networking system are varied but likely include identifying research collaborators and mentors, reporting on institutional research, and strategic planning for the research mission. A 2017 survey by Online Computer Library Center (OCLC) Research found that additional reasons for having a RIMS include internal tracking, publication compliance and grants or awards management [2].

A primary reason for having a RIMS in place is to learn about individual or institutional research output. This allows faculty, staff and students as well as interested external inquirers to gain an understanding of the research initiatives at that organization. In 2012, Boland et al. conducted an analysis of RIMS usage at Columbia University. They found that searchers located researcher profiles much faster in a RIMS when compared to Google and spent more time engaged with the RIMS than Google [3].

Over the last few years, the Penn State CoM RIMS team has presented aspects of their system at research networking, library and translational science conferences. Additionally, the team has assisted other medical school RIMS committees by answering specific questions about their system configuration. Most of these committees were comprised of medical librarians. The goal of this publication is to summarize the queries the team has received and our responses to highlight system considerations for current or future RIMS applications and assist medical librarians involved in these initiatives.

The Penn State RIMS can be viewed publicly [4]. The authors' intentions in sharing details of the RIMS are not to serve as an endorsement or criticism of any one type of system or vendor but rather to highlight features and details of a working system.

### Stakeholders and Teams

Launching or maintaining a RIMS will ideally have broad stakeholder participation to maximize relevance for all entities. Research and education administrators along with other units may have specific uses for a RIMS. While librarians will be familiar with the publication output data, IT specialists may play a role with establishing or updating a RIMS, and in programming to extract or input data via application programming interfaces (APIs) for use in other applications. Vardell et al. describe the implementation of a research networking system in the Miller School of Medicine at the University of Miami by noting a collaborative effort between the library and the Office of Research [5]. The partnership formed between these entities resulted in the library being responsible for marketing and training of the product while the Office of Research maintained technical features.

Penn State maintains a single RIMS, which was established with the goal of representing researchers across all campuses and disciplines to advance interdisciplinary research. Since the RIMS includes over 5000 faculty across 24 campuses and more than a dozen academic colleges, it is managed by two teams: one from the College of Medicine (with over 1500 profiles) and another from University Park (flagship campus). The Penn State CoM RIMS team is comprised of a Medical Librarian, a Multimedia Specialist from the Office of Strategic Marketing and Communications, an Assistant Director of Research Development and the Associate Dean for Interdisciplinary Research. The CoM team is responsible for College of Medicine data while working collaboratively with the University Park team, who maintain the remainder of data from other colleges and campuses across Penn State [6].

Librarians can be meaningful collaborators in RIMS operations, primarily due to their in depth understanding of bibliometrics analysis and publications and can also assist with the maintenance and expansion of RIMS. Research administrators share their knowledge of the research enterprise and have access and knowledge to clean up the often incomplete and incorrect data on faculty researchers. Research administrators benefit from a quality RIMS to collect research data, identify collaborators and mentors for research faculty, help connect students or trainees to research supervisors, and to engage appropriate reviewers for internal award programs and limited submission opportunities. The availability of faculty researcher profiles in the RIMS allows marketing and communication team members to employ direct links to relevant profiles on websites, in news stories, and in other materials about a researcher's projects, grants or initiatives.

### Penn State RIMS Features

Reporting on output by individual researcher and academic unit is the primary utility of the Penn State RIMS and, therefore, requires a significant amount of attention. The RIMS was originally set up with each campus, college and department represented in the hierarchy. Over time, clinical divisions, academic centers and institutes along with research facilities were added to the existing structure. Initially, human resources data with unique identifiers enabled researchers to be added to the RIMS and affiliated with their respective campus/college/department. As additional tiers were added to the hierarchy, such as clinical divisions and institutes, outreach to units to determine membership became required as those data are often not centrally recorded.

The question of which researchers are given profiles does not always have an easy answer. Most universities employ faculty with a range of titles and roles. The overall determining factor in the Penn State RIMS is having a research-intensive position that generates research output. The finer points of faculty inclusion may vary by college or department. Over time, issues such as reaching a maximum profile limit likely will result in modifying criteria for Penn State as its RIMS has a maximum number of allowable profiles. As such, establishing guidelines for profile inclusion and vigilance in removing faculty who have left the institution has developed over time.

Publication data in the Penn State RIMS is primarily driven by Scopus (Elsevier) imports via Scopus author IDs. Until new faculty members are established at the university, it may be necessary to assist with Scopus author ID cleaning so that the correct data are imported and associated with their RIMS faculty profile. The imported Scopus data include citation counts and the interface generates an h index on faculty profile pages [7]. Currently PlumX and Altmetric donuts are available for publications in the RIMS and are connected to publications via Scopus data or digital object identifiers (DOIs).

The College of Medicine RIMS team follows unique workflows for updating and editing content. One of the major differences between the CoM data and the rest of Penn State is that College of Medicine faculty profiles are locked for editing so that the CoM RIMS team makes all profile edits and individual faculty members are not directly involved in their updating or maintain their profiles. A collaboration between the CoM RIMS team and the Marketing department generated an opportunity to build the RIMS around institutional branding, creating a consistent look and feel while maintaining data quality. The Marketing department hosts a form <https://research.med.psu.edu/pure/> for faculty or their proxies to use to update profile data [8]. In this way, the faculty are able to highlight what they deem to be important aspects of their work.

While Scopus captures the majority of biomedicine publications of the faculty, some publications need to be manually entered by the CoM RIMS team. Non-Scopus indexed publications are provided by the faculty using the update form and are manually added to the RIMS to appear in the faculty member's profile. Non-Scopus publications, however, do not display citation counts from Scopus as those publications are not indexed there. To allow faculty to have more comprehensive and forward-looking content that may not be part of their publication history, text boxes for narratives describing researchers' current and future research, clinical and teaching interests are available and faculty members write their narratives and submit them via the RIMS form.

Awards and education and training can also be provided by the researchers. Awards (prizes) are listed in profiles, and the CoM RIMS team allots eight awards per faculty member to limit manual data entry by the RIMS team. Grants and projects are also part of the Penn State RIMS and are auto populated from Elsevier's database. The team is not able to add internal grants at this time due to the amount of manual labor it would require so the grants and projects tab highlights only external funding that is monitored by Elsevier.

To keep the data as up to date as possible, the CoM team conducts monthly profile additions and deletions from the RIMS based on personnel data from the dean's office. The team also implements annual updates to acknowledge promotions and tenure. The task of updating the ranks (e.g. assistant to associate professor) is performed in bulk at their effective date, whereas title changes for leadership roles are done on an ongoing basis. Additional collaborations include quarterly updates from the Office of Development on researchers holding endowed titles. New or updated information provided by faculty occurs on a continuous basis, including changes to narratives or adding an award.

As a member of the RIMS team, the medical librarian plays a significant role in maintaining profiles, performing edits, adding and deleting profiles and consulting about current and future features of the RIMS. It is difficult to estimate the amount of time required in this role, as it varies over time. One example is requiring more time in the summer to create profiles when more hiring takes place. Also, with the change of the fiscal year in July, faculty promotions go into effect, requiring many title changes. Together, the four-member CoM RIMS team likely invests an average of 15 hours a week to manage the resource at its current state.

### Persistent Identifiers in a RIMS

The Penn State College of Medicine Biomedical Research Core Facilities are institution-funded services which require assessment pertaining to their utility as well as marketing of the services to encourage usage. The CoM RIMS team worked with the Director of the Core Facilities to add those units to the university hierarchy in the RIMS, with the desired outcome of featuring relevant publications resulting from core services. The goal of the initiative was to publicly showcase products of the tools and methods from the core facilities, encouraging additional collaborations among researchers. Furthermore, equipment is listed on core facility RIMS profiles so researchers can see the available tools and services. Relevant core facility publications are being captured through the use of Research Resource Identifiers, or RRIDs, and associated with the units.

The use of persistent identifiers in biomedical research and publications to enhance research reproducibility has been on the rise; and assigning RRIDs to equipment, and in this case facilities, is an example of this practice [9]. Researchers include RRIDs in their publications when citing resources such as antibodies, cell lines and select research equipment. To capture CoM core facility publications, the medical librarian on the RIMS team created search strategies comprised of RRIDs, core facility names and the institution name to query Scopus (Elsevier) and Dimensions (Digital Science) databases. A multi-database approach was needed to capture this data, as RRIDs and associated core facility data may be located in the funding section or body of the publication text. Alerts of new publications from the databases are delivered to the core facility manager and librarian on a recurring basis, and the relevant publications are assigned to the appropriate core facility within the RIMS (example of Cryo EM facility <https://pure.psu.edu/en/organisations/cryoem-andcryoet-com-biomedical-core-facility>). The efforts of the CoM RIMS team collaborating with the director of the biomedical core facilities have improved internal return-on-investment analyses while publicly demonstrating applications from research core usage on the RIMS. As uptake of RRID usage increases, reporting on the impact of individual core facilities may be simplified to searches employing only RRIDs.

ORCiD iDs are another example of a persistent identifier that can be integrated into a RIMS [10]. The Penn State RIMS allows researchers to have a hyperlink to their ORCiD iD in their profiles, although, the RIMS is not currently configured for direct export to and import from ORCiD. However, this functionality is available, as Research Triangle International in North Carolina requires the creation of ORCiD iDs that are linked with RIMS profiles to assist with the administrative processes associated with grant applications, as well as connecting associated publications with researchers [11].

### Data Reuse and Reporting

A RIMS can ease the burden of finding and cleaning publication data. Specialized reporting on research output and other data by unit can aid in internal and external reporting, accreditation reporting, as well as helping with metadata reuse.

One example is the Liaison Committee on Medical Education (LCME) accreditation standards among medical school programs. Required data include collecting research output data by department such as number of peer reviewed articles and books/book chapters [12]. Since each new faculty member in the RIMS is assigned to their appropriate department(s) and other units, it is possible to extract department-level data. The librarian on the College of Medicine RIMS team was approached to provide research output data by department, which was made much easier by having a system in place designed to host these data.

Running unit reports on publication output is another area where a librarian can be an asset to the RIMS team. Unit heads or administrative staff frequently approach the CoM RIMS team to run reports on research output from the RIMS. The librarian on the RIMS team has established a sustainable workflow to manage these requests. Upon request, the librarian exports an up-to-date list of Scopus author IDs by unit from the RIMS and conducts one on one Scopus training with the requester using Scopus author IDs from the unit to be queried. This approach has empowered several individuals to set up alerts of interest and provides the ability to run reports as needed without having to ask the RIMS team for every request. Thus, the efforts the team makes to clean Scopus author IDs and to assign the faculty and their IDs to the correct units in the RIMS feeds forward for accurate internal reporting. Rarely does the team provide the manually entered non-Scopus indexed publications for these reports as these publications are a considerable minority, but it is possible to do an export for a requester if that need arises.

Metadata reuse from a RIMS and librarian involvement may be possible for initiatives such as dashboards and use on websites. A 2021 OCLC case study of five US institutions showed metadata reuse from RIMS in various stages of operation, with two reusing metadata and others planning use [6]. In the case of Penn State, the RIMS provides publication and researcher profile data for reuse on the Penn State Cancer Institute website [13]. (sample profile <https://cancer.psu.edu/researchers/individual/-/researcher/5B6500F63D2438DBE0540010E056499A/jay-raman-md>).

Reuse of RIMS data within the College of Medicine is also demonstrated with an internal college-specific administrative dashboard. The dashboard integrates various data sources, including research output from the RIMS, teaching statistics from internal systems, and grants and projects data from other Penn State sources. The librarian on the RIMS team meets with IT and research administrators to clarify the types of data available for hosting on the dashboard and serves as a liaison between the RIMS host (Elsevier) and the academic IT team for issues like API keys and clarification about data structure.

## CONCLUSION

The many roles that medical librarians play in maintaining research networking systems underscore the necessity of integrating librarians within all of the missions of academic health system. On a RIMS team, librarians will assist with explaining aspects of scholarly communications, describe database coverage (e.g. Scopus) and play key roles in data export. Having a team of diverse, interprofessional stakeholders able to share the maintenance and assist in developing future initiatives, ensures that the goals of the networking system are met as well as a robust return on investment.
